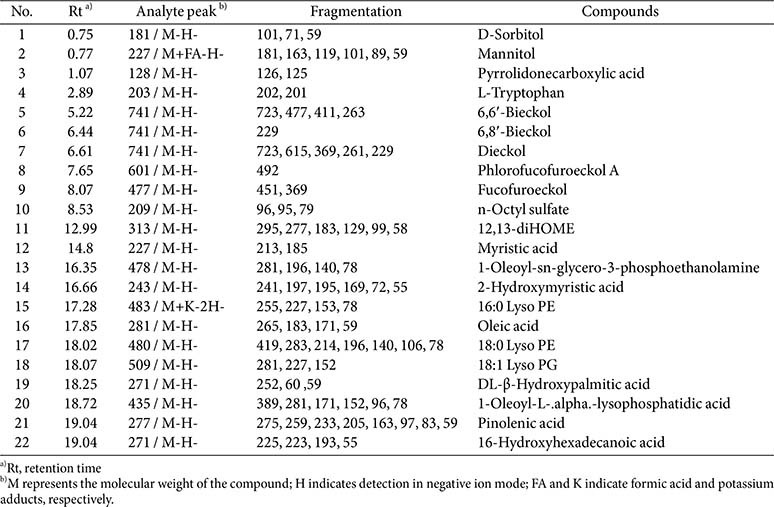# Bioactive Polysaccharides and Phlorotannins from *Eisenia bicyclis* Alleviate Particulate Matter (PM)_2.5_-Induced Chronic Lung Injury by Regulating Inflammatory and Fibrotic Pathways

**DOI:** 10.4014/jmb.2510.10053

**Published:** 2025-12-19

**Authors:** Jong Min Kim, Tae Yoon Kim, Hyo Lim Lee, Ho Jin Heo

**Affiliations:** 1 Division of Applied Life Science (BK21), Institute of Agriculture and Life Science, Gyeongsang National University, Jinju 52828, Republic of Korea; 2 Aging Research Group, Korea Food Research Institute, Wanju 55365, Republic of Korea

**Keywords:** *Eisenia bicyclis*, particulate matter, polysaccharides, phlorotannins, Nrf2 pathway, pulmonary fibrosis

## Abstract

This study investigated the protective effects of the ethanolic extract of *Eisenia bicyclis* (EB) against chronic pulmonary toxicity induced by particulate matter (PM_2.5_) exposure in mice. EB contains phlorotannins, including dieckol and phlorofucofuroeckol A, sulfated polysaccharides, and lipid and amino acid derivatives. Male BALB/c mice were exposed to aerosolized PM_2.5_ for 5 h daily over a repeated-dose period, and EB was administered orally. The administration of EB significantly ameliorated PM_2.5_-induced lung damage by restoring antioxidant defense systems and decreasing serum levels of inflammatory cytokines. EB also suppressed mitochondrial dysfunction and apoptotic signaling. Furthermore, EB attenuated the expression of inflammatory markers, including TLR4, TNF-α, IL-1β, and COX-2, and attenuated the activation of fibrotic signaling pathways via the TGF-β/Smad axis. *In vitro* experiments using A549 cells further supported these findings, demonstrating that EB and its phlorotannin components (dieckol, 6,6'-bieckol, and 6,8'-bieckol) restored cell viability, reduced inflammatory cytokines, and modulated the Nrf2/HO-1 pathway together with fibrotic genes and proteins. These findings suggest that EB, which contains bioactive compounds, may be a promising functional material candidate for mitigating chronic lung injury caused by environmental toxicants such as PM_2.5_.

## Introduction

Particulate matter (PM_2.5_) is one of the main components of air pollution and is an environmentally toxic substance that seriously threatens lung health [1]. PM_2.5_ consists of fine particles with a diameter of 2.5 μm or less and can penetrate deep into the alveoli of the respiratory tract, causing chronic lung tissue damage [2]. In particular, PM_2.5_ contains various toxic compounds such as heavy metals, polycyclic aromatic hydrocarbons (PAHs), and reactive oxygen species (ROS) inducers, which act as chemical irritants and inflammatory factors [3]. While immune-cell–mediated responses such as neutrophil influx and macrophage activation occur in the subsequent phases of PM_2.5_-induced injury, the earliest pathological alterations originate in airway epithelial cells, which initiate oxidative and inflammatory signaling upon direct particle exposure [1]. Long-term exposure to PM_2.5_ continuously induces oxidative stress, inflammatory responses, apoptosis, and tissue remodeling in lung tissue, leading to decreased lung function [4]. Although neutrophil and macrophage recruitment are well-recognized aspects of PM_2.5_-induced lung injury reported in previous studies, the present work focuses on epithelial-cell–driven mechanisms because airway epithelial cells represent the first physical and immunological barrier that directly encounters inhaled PM_2.5_. Regarding molecular mechanism, PM_2.5_ activates the innate immune receptor toll-like receptor 4 (TLR4), which stimulates nuclear factor-κB (NF-κB) and mitogen-activated protein kinase (MAPK) pathways via the myeloid differentiation primary response 88 (MyD88) signaling pathway [5]. This ultimately increases the expression of inflammatory mediators such as tumor necrosis factor-α (TNF-α), interleukin (IL)-1β, and cyclooxygenase-2 (COX-2) [6]. At the same time, the transforming growth factor-β (TGF-β)/Smad signaling pathway is also stimulated, which increases the expression of matrix metalloproteinases (MMPs) such as MMP2 and MMP9 and fibrosis-related genes, thereby promoting fibrosis in lung tissue [7]. Therefore, PM_2.5_-induced pulmonary toxicity involves not only a simple acute inflammatory response but also has a complex pathological mechanism leading to chronic tissue damage and fibrosis [8]. Effective prevention and treatment strategies are required, but treatments for lung damage caused by PM_2.5_ have relied on antioxidants or anti-inflammatory agents. However, long-term administration of antioxidant or anti-inflammatory agents may lead to adverse effects such as immune suppression, gastrointestinal issues, and organ toxicity, which restricts their continued clinical use [9]. Accordingly, natural bioactive materials with safe and multifunctional effects are attracting attention as alternatives. In particular, polysaccharides and phenolic compounds are reported to have various protective effects against oxidative stress, inflammation, and fibrosis, and have the potential to protect lung tissue from chronic environmental toxicity [10]. Therefore, a PM_2.5_ lung toxicity suppression strategy using natural product-based composite materials has value as a new preventive and therapeutic approach.

*Eisenia bicyclis* (EB) is a type of brown algae that has been traditionally used as food or as a medicinal resource [11]. EB is rich in phlorotannins and polysaccharides with structural diversity, and previous studies have reported multiple physiological effects such as antioxidant, anti-inflammatory, and anticancer [12, 13]. In particular, phlorotannins have been suggested to have a strong radical scavenging ability and the ability to regulate signaling pathways related to inflammation and fibrosis, and polysaccharides are also known to have immunomodulatory and tissue-protective activities [14, 15]. However, the physiological effects and mechanisms of action of EB-derived complex components on chronic pulmonary toxicity induced by PM_2.5_ have not been sufficiently studied. Therefore, this study analyzed phlorotannins and polysaccharides from EB to determine whether they showed pulmonary protective effects through antioxidant, anti-inflammatory, and antifibrotic effects in a PM_2.5_-induced lung injury model.

## Materials and Methods

### Sample Preparation

EB was purchased from Wando-gun (Republic of Korea). The EB was thoroughly washed with tap water to remove salts and debris, then lyophilized using a vacuum-tray freeze dryer (Operon, Republic of Korea). The dried sample was ground into powder and independently extracted with 50 volumes of 0, 20, 40, 60, 80, and 95%ethanol at 40°C for 2 h. The extract was filtered through Whatman No. 2 filter paper (Whatman International Limited, UK), concentrated using a rotary vacuum evaporator (N-1000; EYELA Co., Japan), freeze-dried, and stored at −20°C until use. Based on a previous study (Table S1), the 95% ethanolic extract exhibited the highest antioxidant activity among ethanolic extracts. All subsequent experiments were conducted using the 95% ethanolic extract.

### Determination of Molecular Weight

The average molecular weight of the sample was determined using gel permeation chromatography (GPC) with an EcoSEC HLC-8420 GPC system (Tosoh Bioscience, Japan) equipped with a TSKgel guard PWxl, two TSKgel GMPWxl, and a TSKgel G2500PWxl column (7.8 × 300 mm, Tosoh Bioscience). The system was operated with a refractive index (RI) detector and eluted with 0.1 M NaNO_3_ at a flow rate of 1.0 ml/min. The mobile phase and sample were filtered through 0.22 μm nylon membranes before injection. The column temperature was maintained at 40°C, and the injection volume was 100 μl with a sample concentration of 3 mg/ml. Polyethylene glycols/polyethylene oxides standards were used for calibration, and data were processed using EcoSEC Elite-WS software.

### Determination of Total Polysaccharide Content

The total polysaccharide content was quantified by the phenol–sulfuric acid colorimetric method. The sample was reacted with 5% phenol and concentrated sulfuric acid, followed by incubation with gentle shaking for 30 min. The absorbance was then recorded at 490 nm using a microplate spectrophotometer (EPOCH2; BioTek Instruments, USA). A calibration curve was constructed using glucose as the reference standard to determine the final polysaccharide concentration.

### Determination of Sulfate Content

To determine the sulfate content, the sample was first hydrolyzed in 1 M hydrochloric acid at 100°C for 5 h in a water bath. After hydrolysis, the solution was mixed with 3% trichloroacetic acid (TCA) and BaCl_2_-gelatin reagent and incubated for 15 min. The resulting barium sulfate precipitate was measured spectrophotometrically at 360 nm using a microplate reader (EPOCH2; BioTek Instruments). Potassium sulfate was used as a standard to calculate sulfate concentration.

### Determination of Monosaccharide Composition

The monosaccharide composition was analyzed using high-performance anion-exchange chromatography with pulsed amperometric detection (HPAEC-PAD) on a Dionex system (Dionex, USA). Before analysis, the sample was hydrolyzed with trifluoroacetic acid and diluted to a final concentration of 2 mg/ml. Separation was performed using a CarboPac PA1 column (Dionex) with a gradient of 18 mM and 200 mM NaOH. The injection volume was 20 μl, and the flow rate was maintained at 1.0 ml/min

### Bioactive Compound Analysis

For the analysis of major phenolic compounds in EB, we used an HPLC-Ultra Quadrupole Time-of-Flight LC/MS/MS system (X500R, SCIEX, USA) equipped with an ACQUITY UPLC BEH C_18_ column (2.1 × 100 mm, 1.7 μm; Waters Corp., USA). The sample was dissolved in 100% methanol before analysis. The mobile phase consisted of solvent A (water with 0.1% formic acid) and solvent B (acetonitrile with 0.1% formic acid). The flow rate was set at 0.35 ml/min with the following gradient profile: 0.0 min, 100% A; 18.0 min, 20% A and 80% B; 20.0 min, 100% A. The column oven temperature was maintained at 40°C, and the injection was performed in negative ion mode. The mass spectrometry conditions were as follows: drying gas (N_2_) temperature, 120°C; drying gas flow, 30 L/h; nebulizer pressure, 40 psi; capillary voltage, 3.0 kV; fragmentor voltage, 175 V; ramp collision energy, 20–40 V; and mass range, 100–1,500 m/z.

### Animals and Diet

Male BALB/c mice (6 weeks old) were obtained from Samtako Bio Korea (Republic of Korea). The animals were housed under controlled environmental conditions (temperature of 22 ± 2°C, relative humidity of 55%, and a 12 h light/dark cycle). Mice were randomly housed in groups of three to four per cage and allocated into four experimental groups: (1) normal control (NC, exposed to clean air and administered vehicle), (2) PM_2.5_-exposed group (exposed to PM_2.5_ and administered vehicle), and (3–4) PM_2.5_-exposed groups treated with EB at doses of 20 or 40 mg/kg body weight (referred to as EB20 and EB40, respectively). EB was administered orally once daily for 12 consecutive weeks. PM_2.5_ was suspended in purified water and aerosolized into an exposure chamber at a consistent concentration of 500 μg/m^3^ for 5 h daily during the sample treatment period, far exceeding the WHO-recommended 24 h mean exposure limit of 25 μg/m^3^ for humans [16]. The PM_2.5_ (Arizona Test Dust, USA) used in this experiment was previously characterized by Kim *et al*. [17]. It was reported to contain various heavy metals, including Fe (16.91 mg/g), Mg (6.26 mg/g), Mn (0.58 mg/g), Ba (0.22 mg/g), Zn (0.07 mg/g), Cu (0.05 mg/g), Pb (0.03 mg/g), Li (0.02 mg/g), and Cr (0.01 mg/g). At the end of the experimental period, mice were euthanized by CO_2_ asphyxiation, and blood and lung tissues were collected for further analysis. All animal protocols were approved by the Institutional Animal Care and Use Committee of Gyeongsang National University (Approval No. GNU-210803-M0069, approval date: 3 August 2021), and experiments were conducted following institutional ethical guidelines.

### Tissue Preparation

Pulmonary tissues were processed using a bullet blender homogenizer (Next Advance Inc., USA) in either phosphate-buffered saline (PBS) or 10 mM phosphate buffer containing 1 mM EDTA (pH 6.7) at 4°C. Blood samples were centrifuged at 13,000 ×*g* for 10 min at 4°C. The resulting supernatants were collected for further analysis.

### Reduced Glutathione (GSH) Quantification

Tissues were homogenized in phosphate buffer (pH 6.0) and centrifuged at 10,000 ×*g* for 15 min at 4°C. The supernatant was treated with 5% metaphosphoric acid and centrifuged again at 2,000 ×*g*. The resulting supernatants were reacted with 0.26 M Tris-HCl (pH 7.8), 0.65 N NaOH, and o-phthaldialdehyde (1 mg/ml) at 37°C for 15 min. Fluorescence intensity was measured at excitation/emission wavelengths of 320/420 nm using a fluorescence reader (Infinite 200, Tecan Co., Switzerland).

### Superoxide Dismutase (SOD) Activity

For SOD activity analysis, pulmonary tissues were homogenized in PBS and centrifuged at 400 ×*g* for 10 min at 4°C. The resulting pellet was resuspended in assay buffer and centrifuged again at 10,000 ×*g*. SOD activity in the supernatant was then determined using a commercial SOD assay kit (Dojindo Molecular Technologies, Japan) following the manufacturer’s instructions.

### Malondialdehyde (MDA) Quantification

Tissue homogenates prepared in PBS were centrifuged at 2,500 ×*g* for 10 min at 4°C. The supernatants were mixed with 1% phosphoric acid and 0.67% thiobarbituric acid, followed by incubation at 95°C for 1 h. The absorbance of the resulting MDA–TBA complex was measured at 532 nm using a microplate reader (Epoch 2, BioTek Instruments Inc.).

### Serum Inflammatory Biomarkers

To estimate the serum inflammatory cytokines, the IL-1β (MLB00C, R&D Systems), IL-6 (M6000B, R&D Systems), and interferon-γ (IFN-γ) (MIF00, R&D Systems) levels were measured according to the manufacturer's protocols.

### Isolation of Mitochondria from Tissue

Tissue samples were homogenized in 10 volumes of mitochondrial isolation (MI) buffer consisting of 215 mM mannitol, 75 mM sucrose, 20 mM HEPES-Na (pH 7.2), 0.1% BSA, and 1 mM EGTA. The homogenates were first centrifuged at 1,300 ×*g* for 10 min at 4°C to remove nuclei and debris. The resulting supernatants were further centrifuged at 13,000 ×*g* for 10 min at 4°C to pellet mitochondria. The mitochondrial pellets were then resuspended in MI buffer containing 0.1% digitonin and incubated for 5 min to permeabilize membranes. After incubation, 2 ml of MI buffer with 1 mM EGTA was added, followed by centrifugation at 13,000 ×*g* for 15 min at 4°C to isolate the purified mitochondria.

### Measurement of Mitochondrial ROS

Mitochondrial ROS levels were evaluated using 2',7'-dichlorofluorescein diacetate (DCF-DA) in isolated mitochondria. The mitochondrial pellet was suspended in a respiration buffer containing 125 mM KCl, 2 mM KH_2_PO_4_, 20 mM HEPES, 1 mM MgCl_2_, 0.5 mM EGTA, 2.5 mM malate, and 5 mM pyruvate. A final concentration of 25 μM DCF-DA was added, and the mixture was incubated for 20 min at room temperature. Fluorescence intensity was measured at 485 nm excitation and 530 nm emission using a microplate reader (Infinite 200, Tecan).

### Assessment of Mitochondrial Membrane Potential

The mitochondrial membrane potential was measured using JC-1 dye. Isolated mitochondria were incubated with 1 μM JC-1 in MI buffer supplemented with 5 mM pyruvate and 5 mM malate. The mixture was placed in a black 96-well plate and incubated in the dark at room temperature for 20 min. The JC-1 fluorescence was then detected using a microplate reader (Infinite 200, Tecan) at excitation 530 nm and emission 590 nm.

### Measurement of Mitochondrial ATP Production

The intracellular ATP levels were determined using a commercial bioluminescence ATP assay kit (Promega Corp., USA) in accordance with the manufacturer's instructions. Luminescence intensity was measured using a GloMax-Multi+ microplate luminometer (GloMax-Multi+, Promega), and ATP concentrations were calculated based on a standard calibration curve.

### Western Blot Analysis

Pulmonary tissues were first homogenized in a lysis buffer (GeneAll Biotechnology, Republic of Korea) supplemented with 1% protease inhibitor. The homogenates were centrifuged at 13,000 ×*g* for 10 min at 4°C, and the supernatants were collected for subsequent protein analysis. Proteins were separated via SDS-PAGE and transferred to membranes, which were incubated with primary antibodies at 4°C for 12 h. Following this, the membranes were treated with secondary antibodies for 1 h at 25°C. Protein bands were visualized using an iBright Imager (Thermo Fisher Scientific), and their relative densities were quantified with ImageJ software (National Institutes of Health, USA). A list of antibodies used is provided in Supplemental data S1.

### Cell Culture

A549 cells (1 × 10^4^ cells/well) were plated in 96-well plates and allowed to adhere for 24 h. Following incubation, the cells were exposed to PM_2.5_ in the presence or absence of EB, dieckol, 6,6'-bieckol, or 6,8'-bieckol for an additional 24 h. Cell viability was then assessed using the CCK-8 assay kit (DONGIN LS Co., Republic of Korea) in accordance with the manufacturer’s instructions. Absorbance was recorded at 450 nm using a microplate reader (Spectra MAX 190; Molecular Devices, USA).

### Quantitative Real-Time PCR (qRT-PCR)

Total RNA was extracted from A549 human alveolar epithelial cells using an RNeasy Mini Kit (Qiagen, USA) according to the manufacturer’s instructions. The quantity and purity of the isolated RNA were assessed with a NanoDrop spectrophotometer (Thermo Fisher Scientific). Complementary DNA (cDNA) was synthesized from the purified RNA using the iScript cDNA Synthesis Kit (Bio-Rad, USA) with random hexamer primers. Quantitative real-time PCR (qRT-PCR) was performed using the SYBR Green PCR Master Mix (Bio-Rad) in accordance with the standard protocol. The primer sequences used in the analysis were as follows (5' to 3'): MCP-1 forward (F), CAG CCA GAT GCA ATC AAT GCC; MCP-1 reverse (R), TGG AAT CCT GAA CCC ACT TCT G; IL-1β F, ATG ATG GCT TAT TAC AGT GGC AA; IL-1β R, GTC GGA GAT TCG TAG CTG GA; IL-6 F, ACT CAC CTC TTC AGA ACG AAT TG; IL-6 R, CCA TCT TTG GAA GGT TCA GGT TG; Col1A1 F, CAG CCG CTT CAC CTA CAG C; Col1A1 R, TTT CAG AAG GGA AGC AGG GT; TGF-β1 F, GCA ACA TGT GGA ACT CTA CCA GAA; TGF-β1 R, CTG TTG ATG TGG TGT CAG GCA; Fibronectin F, CAG GAG GAA GAA AGG TGG AGC; Fibronectin R, GCT TCC TGT CAG AGG TGA GTT; GAPDH F, ATT GTC AGC AAT GCA TCC TG; GAPDH R, ATG GAC TGT GGT CAT GAG CC. GAPDH served as the endogenous control for normalization.

### Immunofluorescence Assay

A549 cells were seeded at a density of 1 × 10^4^ cells per well and cultured for 24 h to allow cell attachment. After the designated treatments, cells were gently washed with phosphate-buffered saline (PBS) and fixed using 4%paraformaldehyde for 15 min at 25°C. Following fixation, the cells were permeabilized with 0.1% Triton X-100 in PBS for 10 min and subsequently blocked in 5% bovine serum albumin (BSA) for 1 h to prevent nonspecific binding. Cells were then incubated overnight at 4°C with primary antibodies against alpha smooth muscle cells (αSMA, ab5694, Abcam). After thorough PBS washing the next day, samples were treated with a Cy3-conjugated goat anti-rabbit secondary antibody (Servicebio, China) for 2 h at 25°C. Nuclear staining was performed using DAPI (diamidino-2-phenylindole) for 5 min. After final washing with PBS containing 0.05% Tween-20, cells were mounted with VECTASHIELD antifade medium and visualized using an Olympus FY3000 fluorescence microscope (Olympus Co., Japan).

### Statistical Analysis

All results are presented as means ± standard deviations (SD). Statistical comparisons among groups were conducted using one-way analysis of variance (ANOVA). Tukey’s post-hoc test was applied following ANOVA to determine statistical differences among groups. A *p*-value less than 0.05 was considered statistically significant. All analyses were performed using SAS software version 9.4 (SAS Institute Inc., USA). Statistical significance was determined as *p*<0.05 (*), *p*<0.01 (**), or *p*<0.001 (***) compared to the NC group, and as *p*<0.05 (^#^), *p*<0.01 (^##^), or *p*<0.001 (^###^) compared to the PM group.

## Results

### Chemical Composition of Polysaccharide

**Fig. 1:**
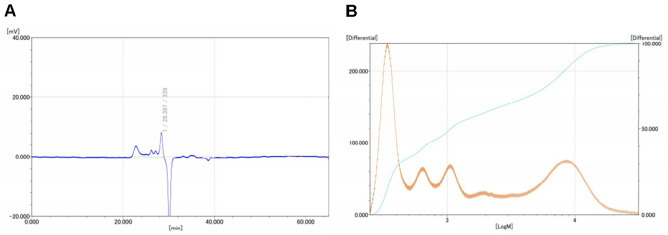
Gel permeation chromatography (GPC) profiles of *Eisenia bicyclis* (EB) ethanolic extract. Molecular weight of peak top at 28.397 min of GPC Chromatogram (**A**). Molecular weight distribution represented as a differential (orange line) and cumulative (blue line) curve plotted against LogM (logarithm of molecular weight).

The structural properties of the polysaccharides extracted from EB were analyzed using various physicochemical techniques. Gel permeation chromatography revealed that EB polysaccharides exhibited a number-average molecular weight (Mn) of 479.33 kDa, a weight-average molecular weight (Mw) of 3,510 kDa, and a peak molecular weight (Mp) of 340.67 kDa, with a polydispersity index (PDI) of 4.68, indicating a heterogeneous molecular weight distribution (Table 1 and Fig. 1). The GPC chromatogram (Fig. 1A) showed a distinct peak at 28.397 min, corresponding to a peak molecular weight of 3,547 kDa. The molecular weight distribution curve (Fig. 1B) demonstrated a polydisperse profile with multiple molecular species, as evidenced by several differential peaks, confirming the heterogeneity of EB polysaccharides.

Polysaccharide and composition of EB were presented in Table 1. The total polysaccharide content in the extract was 11.95%, and the sulfate content was relatively high at 34.10%, indicating that the extract contained sulfated polysaccharides commonly observed in marine-derived species [18]. Monosaccharide composition analysis revealed that the polysaccharides were mainly composed of glucose (47.06%), xylose (20.99%), galactose (19.40%), arabinose (10.59%), and a small amount of rhamnose (1.95%). These sugar residues are typical of heteropolysaccharides from brown algae and are known to contribute to biological activities such as antioxidant, immunomodulatory, and anti-inflammatory functions [19, 20].

### Bioactive Compounds in EB

**Fig. 2:**
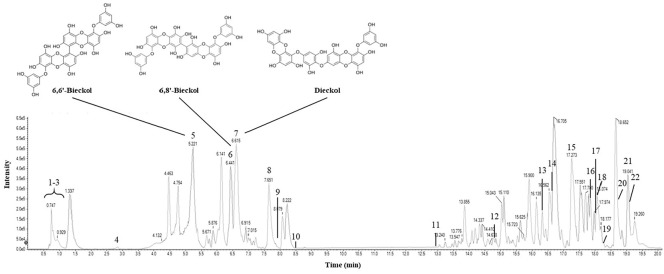
HPLC-Q-TOF-MS chromatogram of *Eisenia bicyclis* (EB) ethanolic extract.

To identify the bioactive compounds in the EB, LC-MS analysis was conducted in negative ion mode (Table 2 and Fig. 2). Twenty-two compounds were tentatively identified based on their retention time (Rt), molecular ion peaks, and fragmentation patterns. The results revealed that EB contains many physiologically active substances, including phlorotannins, sugar alcohols, amino acids, fatty acids, and lipid derivatives. Phlorotannins, which are polyphenolic compounds specific to brown algae, were prominently detected, including 6,6'-bieckol (Rt 5.22 min), 6,8'-bieckol (6.44 min), dieckol (6.61 min), phlorofucofuroeckol A (7.65 min), and fucofuroeckol (8.07 min). Several sugar alcohols, such as D-sorbitol (0.75 min, 181 m/z) and mannitol (0.77 min, 227 m/z), as well as amino acid derivatives like L-tryptophan (2.89 min, 203 m/z) and pyrrolidonecarboxylic acid (1.07 min, 128 m/z), were identified. Moreover, multiple lipid-related metabolites, including fatty acids, such as oleic acid (17.85 min, 281 m/z), myristic acid (14.80 min, 227 m/z), pinolenic acid (19.04 min, 277 m/z), DL-β-hydroxypalmitic acid (18.25 min, 271 m/z), 1-oleoyl-L-alpha-lysophosphatidic acid (18.72 min, 435 m/z), and 16-hydroxyhexadecanoic acid (19.04 min, 271 m/z), and lysophospholipids, such as 16:0 Lyso PE (17.28 min, 483 m/z), 18:0 Lyso PE (18.02 min, 480 m/z), and 18:1 Lyso PG (18.07 min, 509 m/z) were detected.

### Antioxidant Biomarkers

**Fig. 3:**
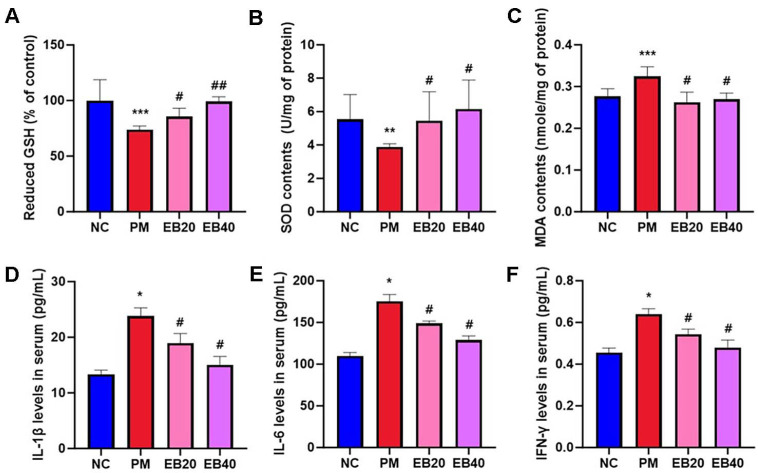
Protective effect of ethanolic extract of *Eisenia bicyclis* (EB) on reduced glutathione level (A), SOD content (B), and MDA content (C) in pulmonary tissue. Results shown are mean ± SD (n = 5). Data were considered statistically significant at *p* < 0.05. *p < 0.05, **p < 0.01, ***p < 0.001, compared to the NC group. #*p* < 0.05, ##*p* < 0.01, ###*p* < 0.001, compared to the PM group.

Reduced pulmonary GSH levels and SOD activity are presented in Fig. 3A and 3B. The PM group exhibited a marked reduction in GSH content and SOD activity (73.95% and 3.88 U/mg of protein, respectively) compared to the NC group (100.00% and 5.54 U/mg of protein, respectively). However, EB-treated groups showed a noticeable improvement. In the EB20 and EB40 groups, GSH levels and SOD activity were restored to 85.51% and 5.46 U/mg of protein, and 99.03% and 6.16 U/mg of protein, respectively.

Pulmonary MDA concentration is shown in Fig. 3C. In the PM group, MDA level increased to 0.32 nmole/mg of protein, whereas those in the NC group were 0.28 nmole/mg of protein. Treatment with EB significantly attenuated these changes. The EB groups showed values of 0.26 and 0.27 nmole/mg of protein, respectively, indicating protective effects against PM_2.5_-induced oxidative stress.

### Inflammatory Cytokines in Serum

Inflammatory cytokines in serum are presented in Fig. 3D-3F. The PM group exhibited elevated expression of IL-1β (23.82 pg/ml), IL-6 (175.27 pg/ml), and IFN-γ (0.64 pg/ml) compared to the NC group (13.34 pg/ml, 109.91 pg/ml, and 0.45 pg/ml, respectively). In contrast, EB20 and EB40 treatment significantly downregulated IL-1β (18.93 and 15.06 pg/ml), IL-6 (149.00 and 128.89 pg/ml), and IFN-γ (0.54 and 0.48 pg/ml) levels.

### Mitochondrial Function

**Fig. 4:**
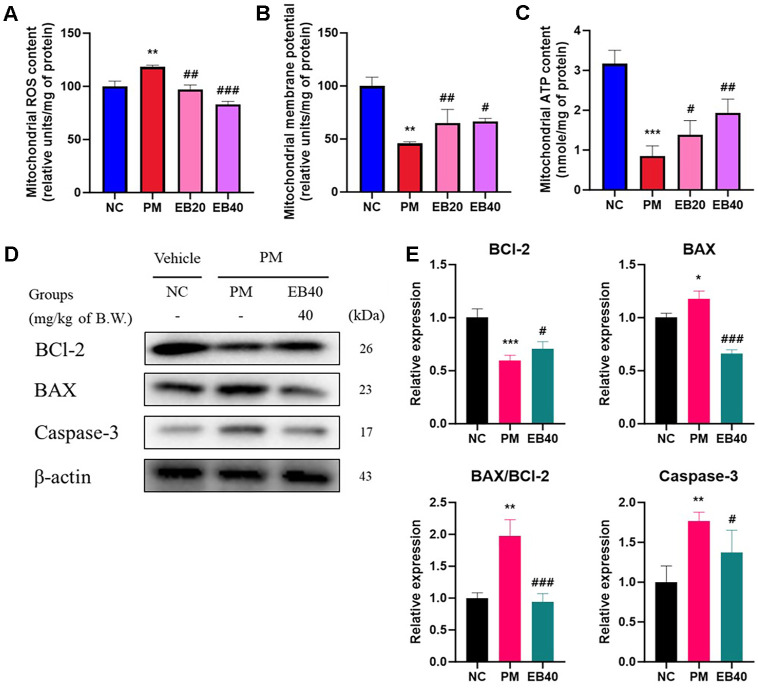
Protective effect of ethanolic extract of *Eisenia bicyclis* (EB) on mitochondrial ROS content (A), mitochondrial membrane potential (MMP) level, mitochondrial ATP content (C), western blot band images (D). Protein expression levels related to mitochondrial apoptic signal (E) in pulmonary tissues. Results shown are mean ± SD (n = 3). Data were considered statistically significant at *p* < 0.05. *p < 0.05, **p < 0.01, ***p < 0.001, compared to the NC group. #*p* < 0.05, ##*p* < 0.01, ###*p* < 0.001, compared to the PM group.

Mitochondrial ROS levels are shown in Fig. 4A. The PM group exhibited a significant increase in mitochondrial ROS production (118.46% of relative units/mg of protein) compared to the NC group (100% of relative units/mg of protein), indicating oxidative stress within mitochondria. However, EB treatment significantly reduced ROS production (97.08 and 83.15% of relative units/mg of protein) against PM_2.5_-induced damage. The results of the mitochondrial membrane potential and ATP content are presented in Fig. 4B and 4C. The PM group showed a significant decline in the mitochondrial membrane potential levels (45.72% of control) and ATP content (0.86 nmole/mg of protein) compared to the NC group (100.00% and 3.17 nmole/mg of protein). In contrast, the EB20 and EB40 groups preserved the mitochondrial membrane potential levels (65.17 and 66.68% of the NC group) and ATP content (1.39 and 1.94 nmole/mg of protein), respectively.

### Mitochondrial Protein Expression Level in Lung Tissue

Protein expressions related to mitochondrial apoptosis are shown in Fig. 4D and 4E. The expression levels of BAX (117.85%), the BAX/BCl-2 ratio (1.98), and caspase-3 (176.81%) were increased in the PM group compared to the NC group, indicating the activation of mitochondrial apoptosis. In contrast, EB40 supplementation significantly decreased BAX expression (66.25%), BAX/BCl-2 ratio (0.94), and caspase-3 expression (137.31%). BCl-2 expression was reduced in the PM group (59.70%) but was restored in the EB40 group (70.60%).

### Inflammatory and Fibrosis Protein Expression Level in Lung Tissue

**Fig. 5:**
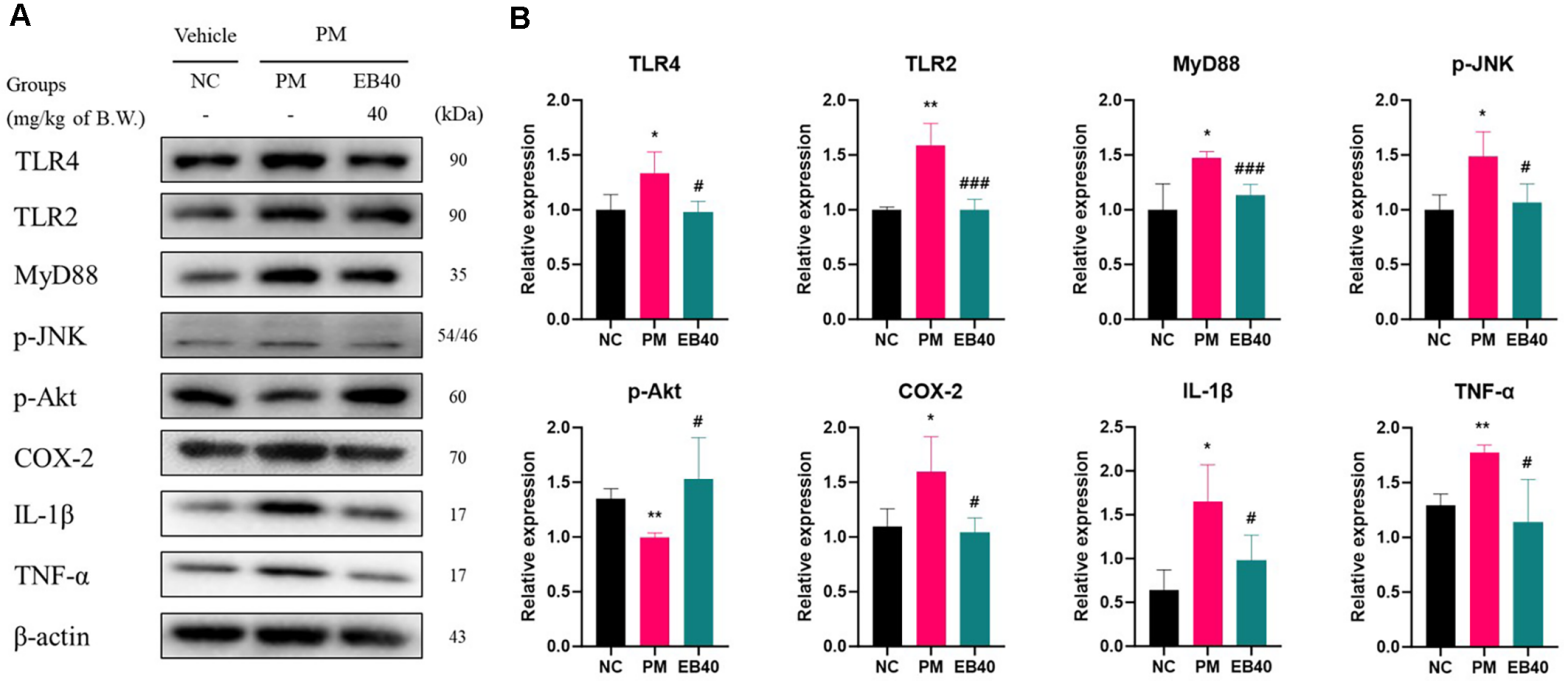
Protective effect of ethanolic extract of *Eisenia bicyclis* (EB) on inflammatory protein expression. Western blot band images (A). Protein expression levels related to inflammation (B) in pulmonary tissues. Results shown are mean ± SD (n = 3). Data were considered statistically significant at *p* < 0.05. *p < 0.05, **p < 0.01, ***p < 0.001, compared to the NC group. #*p* < 0.05, ##*p* < 0.01, ###*p* < 0.001, compared to the PM group.

Protein expressions related to inflammatory signaling are presented in Fig. 5. The PM group exhibited elevated expression of TLR4 (133.60%), TLR2 (158.35%), MyD88 (148.61%), p-JNK (148.80%), COX-2 (159.73%), IL-1β (165.29%), and TNF-α (137.53%) compared to the NC group. In contrast, EB40 treatment significantly downregulated TLR4 (98.07%), TLR2 (99.75%), MyD88 (113.53%), p-JNK (106.45%), COX-2 (104.43%), IL-1β (98.54%), and TNF-α (88.40%) levels. p-Akt expression was reduced in the PM group (73.64%) but was restored in the EB40 group (113.37%).

**Fig. 6:**
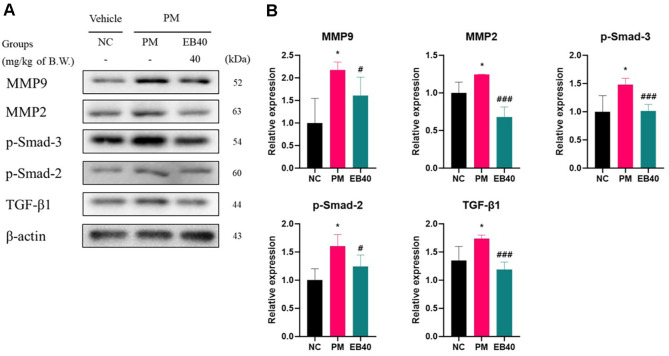
Protective effect of ethanolic extract of *Eisenia bicyclis* (EB) on fibrosis protein expression. Western blot band images (**A**). Protein expression levels related to fibrosis (**B**) in pulmonary tissues. Results shown are mean ± SD (n = 3). Data were considered statistically significant at *p* < 0.05. *p < 0.05, **p < 0.01, ***p < 0.001, compared to the NC group. #*p* < 0.05, ##*p* < 0.01, ###*p* < 0.001, compared to the PM group.

Fibrosis-related protein expressions are shown in Fig. 6. The levels of MMP9 (217.51%), MMP2 (124.51%), p-Smad-3 (148.24%), p-Smad-2 (160.73%), and TGF-β1 (173.69%) were markedly increased in the PM group, indicating fibrotic remodeling. However, EB40 administration significantly reduced the expression of MMP9 (161.24%), MMP2 (67.96%), p-Smad-3 (101.63%), p-Smad-2 (124.50%), and TGF-β1 (118.79%) compared to the PM group.

### Cell Viability and Expression Levels of mRNA and Protein in A549 Cells

**Fig. 7:**
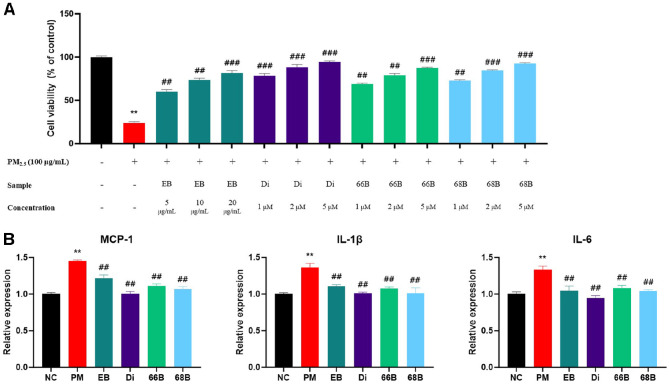
Protective effect of *Eisenia bicyclis* (EB) and its phlorotannin derivatives, including dieckol (Di), 6,6'- bieckol (66B), and 6,8'-bieckol (68B), on cell viability and fibrosis-related signaling in PM_2.5_-treated A549 cells. Cell viability was assessed by CCK-8 assay (**A**). Relative mRNA expression levels of inflammatory markers MCP-1, IL- 1β, and IL-6 (**B**). Data were considered statistically significant at *p* < 0.05. *p < 0.05, **p < 0.01, ***p < 0.001, compared to the NC group. #*p* < 0.05, ##*p* < 0.01, ###*p* < 0.001, compared to the PM group.

Cell viability and molecular changes induced by PM_2.5_ exposure in A549 cells are presented in Fig. 7. As shown in Fig. 7A, PM_2.5_ exposure significantly reduced cell viability (23.81%) compared to the control group, indicating cytotoxicity of fine dust. However, treatment with 20 μg/ML of EB (81.59%), 5 μM dieckol (Di, 94.51%), 5 μM 6,6'-bieckol (66B, 87.42%), or 5 μM 6,8'-bieckol (68B, 92.54%) restored cell viability in a dose-dependent manner.

mRNA expression levels related to inflammatory signaling are presented in Fig. 7B. The PM group showed increased expression of MCP-1 (145.23%), IL-1β (136.44%), and IL-6 (133.33%) compared to the untreated control, indicating activation of inflammatory responses in A549 cells. In contrast, treatment with EB markedly reduced MCP-1 (121.67%), IL-1β (110.73%), and IL-6 (104.65%) expression levels. Di (100.22%, 101.03%, and 94.60%, respectively), 66B (111.43%, 107.56%, and 108.38%, respectively), and 68B (106.64%, 101.12%, and 104.47%, respectively) also showed suppression of inflammatory responses.

**Fig. 8:**
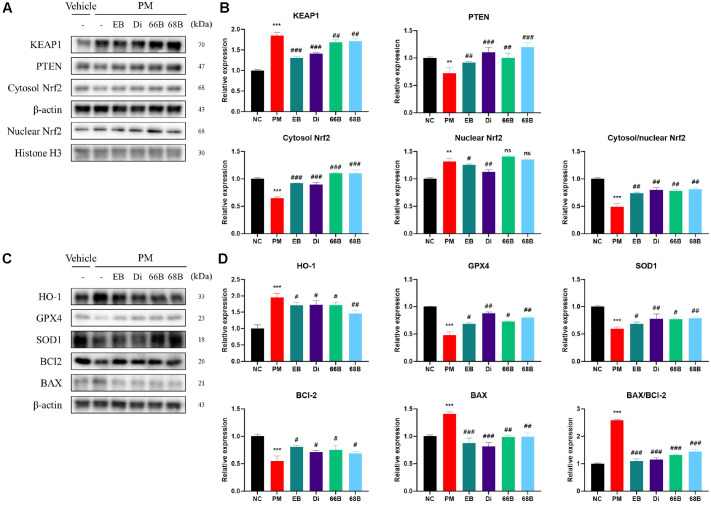
Protective effect of *Eisenia bicyclis* (EB) and its phlorotannin derivatives, including dieckol (Di), 6,6'-bieckol (66B), and 6,8'-bieckol (68B), on Nrf2 and HO-1/apoptosis pathway in PM_2.5_-treated A549 cells. Western blot band images (**A**) and densitometric quantification (**B**) of p-Smad- 3, p-Smad-2, TGF-β1, and αSMA protein expression in A549 cells. Western blot band images (**C**) and densitometric quantification (**D**) of HO-1, and GPX4, SOD1, BCl-2, and BAX protein expression in A549 cells. Results shown are mean ± SD (n = 3). Data were considered statistically significant at *p* < 0.05. *p < 0.05, **p < 0.01, ***p < 0.001, compared to the NC group. #*p* < 0.05, ##*p* < 0.01, ###*p* < 0.001, compared to the PM group.

Protein expressions related to the Nrf2 pathway are shown in Fig. 8A and 8B. The PM group showed increased expression of KEAP1 (184.97%) compared to the untreated control in A549 cells. In contrast, treatment with EB markedly reduced KEAP1 (130.57%) expression level. Di (140.57%), 66B (168.79%), and 68B (171.57%) also showed inhibitory effects. The PM group showed decreased expression of PTEN (72.14%) and ratio of cytosol/nuclear Nrf2 (49.14%) compared to the untreated control in A549 cells. In contrast, treatment with EB markedly increased PTEN (91.87%) expression level and cytosol/nuclear Nrf2 (73.67%) ratio. Di (110.02% and 79.75%, respectively), 66B (110.28% and 78.35%, respectively), and 68B (120.11% and 81.39%, respectively) also showed regulatory effects on the Nrf2 pathway.

Protein expressions related to the HO-1/apoptosis pathway are shown in Fig. 8C and 8D. The PM group showed increased expression of HO-1 (195.71%) and BAX/BCl-2 ratio (258.57%) compared to the untreated control in A549 cells. In contrast, treatment with EB markedly reduced of the HO-1 (170.27%) expression level and BAX/BCl-2 ratio (108.79%). Di (172.10% and 115.52%, respectively), 66B (170.88% and 131.57%, respectively), and 68B (145.76% and 144.35%, respectively) also showed inhibitory effects. The PM group showed decreased expression of GPX4 (48.17%) and SOD1 (59.87%) compared to the untreated control in A549 cells. In contrast, treatment with EB markedly increased GPX4 (68.69%) and SOD1 (68.68%) expression levels. Di (87.93% and 77.80%, respectively), 66B (72.83% and 77.08%, respectively), and 68B (80.07% and 78.87%, respectively) also showed regulatory effects on the HO-1/apoptosis pathway.

### mRNA and Protein Expressions and Immunofluorescence Stain in A549 Cells

**Fig. 9:**
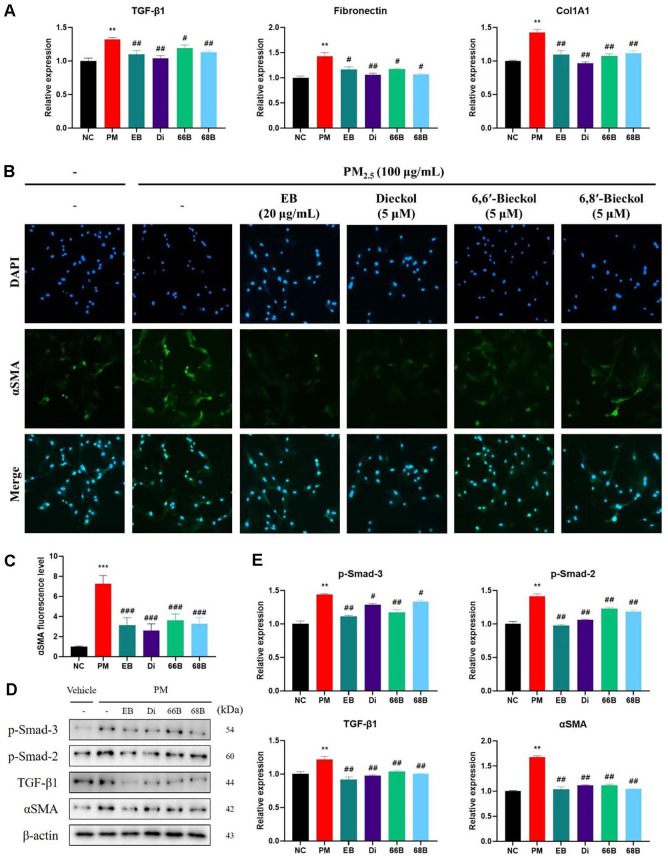
Protective effect of *Eisenia bicyclis* (EB) and its phlorotannin derivatives, including dieckol (Di), 6,6'-bieckol (66B), and 6,8'-bieckol (68B), on αSMA expression in PM_2.5_-treated A549 cells. mRNA expression levels of fibrotic markers TGF-β1, fibronectin, and Col1A1 (**A**). Representative immunofluorescence images showing αSMA (green) and DAPI-stained nuclei (blue). Scale bar = 100 μm (**B**). Fluorescence levels of αSMA (**C**). Western blot band images (**D**) and densitometric quantification (**E**) of p-Smad-3, p-Smad-2, TGF-β1, and αSMA protein expression in A549 cells. *p < 0.05, **p < 0.01, ***p < 0.001, compared to the NC group. #*p* < 0.05, ##*p* < 0.01, ###*p* < 0.001, compared to the PM group.

Fibrosis-related mRNA expression is shown in Fig. 9A. The PM group exhibited increased expression of TGF-β1 (131.87%), fibronectin (142.54%), and Col1A1 (142.31%), suggesting pro-fibrotic remodeling. These increases were significantly attenuated in cells treated with EB (TGF-β1, 109.92%; fibronectin, 116.90%; Col1A1, 109.72%) and its components. Di (104.13%, 106.27%, and 96.29%, respectively), 66B (118.83%, 117.29%, and 107.42%, respectively), and 68B (113.17%, 106.49%, and 111.47%, respectively) further suppressed the expression of these fibrosis-associated genes.

The immunofluorescence results for αSMA expression in A549 cells are shown in Fig. 9B and 9C. Compared to the untreated control, exposure to PM_2.5_ markedly increased the green fluorescence signal intensity (376.65%), indicating enhanced expression of αSMA, a key marker associated with epithelial-to-mesenchymal transition and fibrotic activation. However, treatment with EB (95.26%) and its phlorotannin components, such as Di (93.68%), 66B (110.76%), and 68B (119.99%), significantly reduced the αSMA fluorescence signal. Among them, 66B and 68B demonstrated the most pronounced suppression of αSMA expression, suggesting potent anti-fibrotic properties.

Protein expressions related to fibrotic remodeling are shown in Fig. 9D and 9E. The PM_2.5_-exposed group exhibited increased levels of p-Smad-3 (143.61%), p-Smad-2 (141.43%), TGF-β1 (121.92%), and αSMA (167.87%) compared to the untreated control, indicating activation of fibrosis-associated signaling. In contrast, treatment with EB significantly downregulated the expression of p-Smad-3 (111.21%), p-Smad-2 (97.79%), TGF-β1 (91.85%), and αSMA (103.41%). Di (128.96%, 106.01%, 97.78%, and 111.54%, respectively), 66B (117.15%, 122.79%, 103.69%, and 111.67%, respectively), and 68B (133.32%, 118.47%, 100.33%, and 104.09%, respectively) also reduced these markers.

### Comparative Evaluation of EB-Derived Phlorotannin Compounds in A549 Cells

**Fig. 10:**
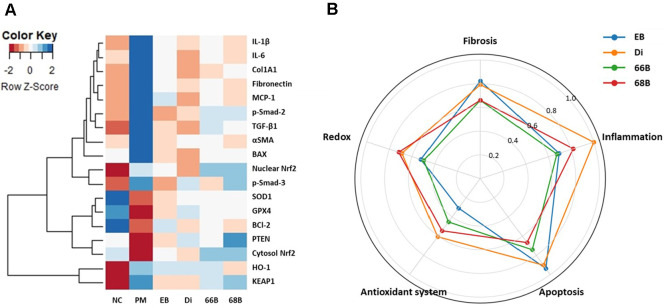
Comparative effect profiling of phlorotannin derivatives, including dieckol (Di), 6,6'-bieckol (66B), and 6,8'-bieckol (68B), in *Eisenia bicyclis* (EB) in PM_2.5_-treated A549 cells. Clustered heatmap visualization showing biomarker expression (**A**). Radar plot analysis illustrating domain-specific recovery scores normalized to NC and PM groups across redox, antioxidant system, apoptosis, inflammation, and fibrosis pathways (**B**).

To further evaluate the differential contribution of individual phlorotannin compounds from EB, biomarker expression profiles were compared in A549 cells following treatment with Di, 66B, and 68B. Hierarchical clustering of multiple markers related to redox regulation, antioxidant enzymes, apoptosis, inflammation, and fibrosis revealed that each compound exerted distinct protective effects. At the same time, EB extract demonstrated a balanced effect across pathways (Fig. 10A). Notably, Di clustered closer to the EB group in antioxidant- and apoptosis-related markers, whereas 66B and 68B showed greater overlap in fibrotic and redox pathways.

Radar plot analysis highlighted these differences more clearly (Fig. 10B). EB treatment maintained relatively even activity across all domains, indicating its broad-spectrum protective potential. In contrast, Di exhibited a pronounced effect on antioxidant enzymes and apoptosis regulation, consistent with its role in mitochondrial stabilization. 66B showed more significant activity in Nrf2-associated redox control, while 68B preferentially modulated HO-1 and fibrotic remodeling. These results suggest that individual phlorotannin compounds possess complementary strengths and that the complex mixture in EB confers a more comprehensive protection against PM_2.5_-induced cellular injury.

## Discussion

This study evaluated the physiological protective effects of EB containing phlorotannins and polysaccharides from EB in a PM_2.5_-induced chronic lung injury model from various perspectives. The experimental results showed that EB alleviates lung tissue damage through various mechanisms, including antioxidant and anti-inflammatory activities, apoptosis regulation, and fibrosis inhibition. These results suggest that EB may act as a bioactive complex that can comprehensively regulate various pathological mechanisms rather than a single functional material. In this section, we compare and interpret these results with previous studies and discuss the mechanistic significance and application potential of EB.

The polysaccharides contained in EB showed a broad molecular weight distribution in GPC analysis, indicating a polydisperse and structurally heterogeneous system (Table 1 and Fig. 1). The marked difference between number- and weight-average molecular weights suggested the dominance of high–molecular-weight fractions, a typical feature of marine-derived sulfated polysaccharides [18]. Monosaccharide composition analysis revealed that galactose and glucose were the major sugars, and the relatively high sulfate content further supported their classification as sulfated polysaccharides. These structures are well known for exerting antioxidant, immunomodulatory, and anti-inflammatory activities through mechanisms such as the inhibition of intracellular ROS, the enhancement of antioxidant enzymes, and the regulation of NF-κB and TLR4 pathways [4, 18]. LC–MS analysis additionally identified several characteristic phlorotannins, including 6,6'-bieckol, 6,8'-bieckol, dieckol, phlorofucofuroeckol A, and fucofuroeckol (Fig. 2) [19]. These compounds exhibit strong radical-scavenging, metal-chelating, and anti-inflammatory activity owing to their multiple hydroxyl groups [20-23]. EB also contained sugar alcohols (D-sorbitol, mannitol), amino acids (L-tryptophan), and various fatty acids and lipid derivatives such as oleic acid, myristic acid, pinolenic acid, and DL-β-hydroxypalmitic acid (Fig. 2), which have been reported to stabilize cell membranes, modulate cytokine secretion, and support tissue regeneration [24-26]. Together, these findings indicate that EB is a complex mixture of polysaccharides, phlorotannins, amino acids, sugar alcohols, and lipid derivatives, each contributing to antioxidant, anti-inflammatory, immunomodulatory, and protective actions. This compositional diversity provides a mechanistic basis for the broad protective effects observed in this study against PM_2.5_-induced pulmonary injury.

PM_2.5_ is sufficiently small to reach the alveoli and carries toxic substances such as heavy metals (Fe, Cu, Pb), PAHs, carbon-centered radicals, and peroxide derivatives on its surface [3]. Once inhaled, these components activate NADPH oxidase, disrupt the mitochondrial electron transport chain, and oxidize membrane lipids, leading to sustained oxidative stress [20, 27]. Chronic exposure further impairs the antioxidant defense systems of alveolar macrophages and epithelial cells, including GSH, SOD, and Nrf2, leading to excessive ROS accumulation, lipid peroxidation, cellular injury, and inflammation [20]. Although HO-1 functions as an antioxidant enzyme, prolonged PM_2.5_ exposure often induces abnormal HO-1 overexpression associated with pathological stress; thus, the EB-mediated reduction observed in this study is interpreted as normalization of dysregulated signaling rather than suppression of protective pathways [6]. PM_2.5_-exposed mice showed increased MDA levels and decreased GSH and SOD activity, confirming oxidative damage in lung tissue (Fig. 3A–3C), whereas EB administration for 12 weeks significantly restored these oxidative indices. LC–MS analysis revealed that EB contains phlorotannins such as 6,6'-bieckol, dieckol, and phlorofucofuroeckol A (Table 2 and Fig. 2), which exhibit potent radical-scavenging and Nrf2-activating properties [27]. EB also includes sulfated polysaccharides that can chelate metal ions and inhibit Fenton chemistry, thereby supporting antioxidant enzyme activity [29]. In addition, sugar alcohols, amino acids, and fatty acids, including D-sorbitol, mannitol, L-tryptophan, oleic acid, myristic acid, DL-β-hydroxypalmitic acid, and pinolenic acid, were detected, which are known to stabilize membranes and modulate oxidative and inflammatory responses [29, 30]. Collectively, these components form a complementary, multi-component antioxidant system, providing a mechanistic basis for EB's broad protective effects against PM_2.5_-induced pulmonary oxidative damage.

Chronic exposure to PM_2.5_ is a major environmental toxic factor that contributes to mitochondrial dysfunction in lung tissue [31]. PM_2.5_ disrupts electron transport chain complexes and induces excessive ROS production, resulting in loss of mitochondrial membrane potential, reduced ATP generation, and opening of the mitochondrial permeability transition pore [27]. These changes trigger cytochrome c release, caspase activation, and an imbalance between BAX and BCL-2, ultimately promoting apoptosis [31]. Consistent with this mechanism, PM_2.5_ exposure significantly increased BAX and cleaved caspase-3 while decreasing BCL-2 in lung tissue (Fig. 4D and 4E). EB administration restored these apoptotic markers, with the EB40 group showing decreased BAX, increased BCL-2, and inhibition of cleaved caspase-3, indicating strong anti-apoptotic activity. This effect is likely associated with the mitochondrial protective actions of phlorotannin derivatives such as 6,6'-bieckol, 6,8'-bieckol, dieckol, phlorofucofuroeckol A, and fucofuroeckol, which have been shown to reduce mitochondrial ROS, maintain membrane potential, and prevent cytochrome c release [32, 33]. Additionally, lipid and amino acid derivatives, including oleic acid, pinolenic acid, and L-tryptophan, identified in EB may contribute to mitochondrial membrane stabilization and anti-apoptotic regulation [34, 35]. Taken together, the multi-component activity of EB appears to play a key role in preserving mitochondrial function and regulating apoptosis signals, thereby alleviating PM_2.5_-induced tissue injury beyond simple antioxidant effects. This protection may arise from upstream modulation of KEAP1, PTEN, and Nrf2 signaling and downstream suppression of TGF-β/Smad activation, αSMA expression, and ECM accumulation, suggesting that EB regulates redox balance and fibrosis pathways in an interconnected manner.

PM_2.5_ is known to activate the innate immune receptor TLR4 through stimulation by LPS-like components, organic compounds, and metal ions attached to the particle itself, which promotes the MyD88-NF-κB pathway and increases the expression of various inflammatory mediators such as TNF-α, IL-1β, and COX-2 [36]. This response leads to chronic inflammation in lung tissue, tissue restructuring, and fibrosis, which are ultimately associated with decreased lung function [37]. In this study, the expression of TLR4, TNF-α, IL-1β, and COX-2 in lung tissue significantly increased in the group exposed to PM_2.5_, which is interpreted as a typical inflammatory response pattern induced by PM_2.5_ (Fig. 5). On the other hand, the expression of the corresponding inflammatory proteins significantly decreased in the group administered EB. The inhibitory effect was particularly evident in the high-dose treatment group (EB40 group). This suggests that EB can effectively control excessive immune response and inflammatory cytokine secretion induced by PM_2.5_. This effect seems to be closely related to the action of phlorotannin derivatives contained in large quantities in EB. According to previous reports, phlorotannins such as dieckol, phlorofucofuroeckol A, and fucofuroeckol have been reported to have anti-inflammatory mechanisms such as inhibition of TLR4 receptor binding, blocking of NF-κB nuclear translocation, and inhibition of pro-inflammatory cytokine expression [38, 39]. In addition, some fatty acid derivatives contained in EB, such as oleic acid and DL-β-hydroxypalmitic acid, are also known to have physiological activities that can control inflammatory responses by suppressing macrophage activity and regulating prostaglandin synthesis and inflammatory response [40, 41]. Therefore, rather than blocking a single inflammatory pathway, EB broadly suppresses inflammatory responses through multiple molecular mechanisms, which increases its applicability as a functional material that responds to complex environmental stimuli such as PM_2.5_.

Long-term exposure to PM_2.5_ induces persistent inflammation and repeated cycles of tissue injury and repair, ultimately leading to fibrosis [8]. PM_2.5_ is known to activate the TGF-β1/Smad-2/3 pathway, promote fibroblast proliferation, and upregulate extracellular matrix (ECM) components such as collagen and fibronectin, while also increasing the activities of MMP2 and MMP9 that accelerate pathological tissue remodeling [42]. Consistent with these mechanisms, expression of TGF-β1, Smad-2/3, and αSMA increased in both lung tissue and A549 cells in the PM_2.5_ exposure group (Figs. 6 and 9). EB administration markedly reduced these fibrosis-related indicators, demonstrating an apparent suppressive effect on PM_2.5_-induced fibrotic activation. In addition, individual phlorotannin constituents of EB—dieckol, 6,6'-bieckol, and 6,8'-bieckol—exhibited direct anti-inflammatory and antifibrotic activities, supporting their contribution to attenuating TGF-β1/Smad signaling (Figs. 8 and 9). Parallel findings in A549 cells, including changes in Nrf2/HO-1, BAX/BCL-2, and antioxidant markers, further substantiated the consistency of EB’s protective actions across models. EB treatment simultaneously suppressed TGF-β1 expression, reduced MMP2/9 activity, and normalized ECM remodeling markers, including Col1A1, fibronectin, and αSMA, confirming its inhibitory effect on PM_2.5_-induced fibrosis. The observed protection is likely mediated by multiple bioactive compounds in EB, including phlorotannins with ROS-reducing and TGF-β1-modulating properties, as well as lipid derivatives such as oleic acid, pinolenic acid, and 1-oleoyl-sn-glycero-3-phosphoethanolamine, which have been reported to have anti-inflammatory and antifibrotic functions [26, 43]. Collectively, these results suggest that EB acts as a multifunctional complex that regulates oxidative stress, inflammation, and aberrant tissue remodeling, thereby mitigating fibrosis associated with chronic PM_2.5_ exposure.

In summary, EB effectively improved various pathological changes in a PM_2.5_-induced chronic lung injury model by maintaining the antioxidant defense system, inhibiting apoptosis, regulating the inflammatory response, and inhibiting fibrosis progression. These results suggest that it is due to the interaction of complex bioactive components, such as phlorotannins, sulfated polysaccharides, fatty acids, and amino acid derivatives in EB. It ultimately has the potential to be used as a functional material for preventing or improving lung diseases caused by environmental toxicity.

## Conclusion

In this study, we used a PM_2.5_-induced chronic lung injury mouse model to evaluate the physiological activity of EB-derived complex extract. EB effectively improved various pathological mechanisms, including the antioxidant defense system, preservation of mitochondrial function, inhibition of apoptosis, inhibition of inflammatory response, and alleviation of fibrosis progression. These results are believed to result from the combined action of various physiologically active components in EB, such as phlorotannins, including dieckol and phlorofucofuroeckol A, sulfated polysaccharides, fatty acids, and amino acid derivatives. EB demonstrates potential as a multifunctional material to combat PM_2.5_-induced pulmonary toxicity, supporting its future application as a functional material candidate. However, this study is limited in confirming the mechanism centered on biochemical and molecular biological indicators based on *in vivo* experiments, and it did not clearly distinguish the mechanistic roles of individual effective ingredients in EB. In addition, there are certain limitations in interpretation because the complex exposure conditions of PM_2.5_ were not fully reproduced. Furthermore, our evaluation relied mainly on molecular and histological markers and did not include direct pulmonary function tests or collagen quantification assays, which may limit functional interpretation. These aspects should be addressed in future studies to substantiate the translational potential of EB. In the future, cell-based mechanism verification at the component fraction or single substance level, multi-organ repeated exposure models, and studies on tracking metabolite changes after ingestion should be conducted in parallel to elucidate the mechanism of EB more precisely and to facilitate its development as a functional agent for environmental toxicity-related lung disease prevention.

## Supplemental Materials

Supplementary data for this paper are available on-line only at http://jmb.or.kr.



## Figures and Tables

**Table 1:** Gel permeation chromatography (GPC), polysaccharide, and composition of ethanolic extract of *Eisenia bicyclis* (EB).

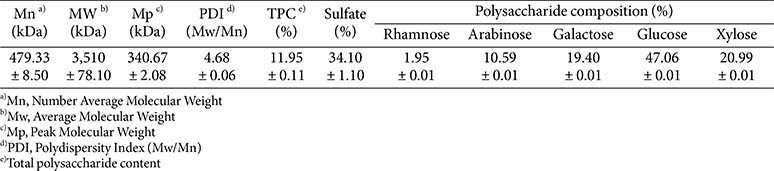

**Table 2:** Identification compounds of ethanolic extract of *Eisenia bicyclis* (EB) by HPLC-MS analysis.