# Supplementary material for: Bioactive Polysaccharides and Phlorotannins from Eisenia bicyclis Alleviate Particulate Matter (PM)2.5-Induced Chronic Lung Injury by Regulating Inflammatory and Fibrotic Pathways
Source: J Microbiol Biotechnol. 2025 Dec 19;35:e2510053. doi: 10.4014/jmb.2510.10053 (PMC12744962; doi:10.4014/jmb.2510.10053)
Supplement: Supplementary file 1 [file jmb-35-e2510053-supple.pdf]

J. Microbiol. Biotechnol. 2025. 35: e2510053

https://doi.org/10.4014/jmb.2510.10053

## Supplementary Table S1. List of antibody details used in this study.

| Antibody | Catalog | Concentration | Manufacture |
|---|---|---|---|
| β-actin | sc-69879 | 1:1,000 | Santa Cruz Biotech. (Dallas, TX, USA) |
| Histone H3 | sc-517576 | 1:1,000 | Santa Cruz Biotech. |
| BCl-2 | sc-509 | 1:1,000 | Santa Cruz Biotech. |
| BAX | sc-7480 | 1:1,000 | Santa Cruz Biotech. |
| TLR4 | sc-52962 | 1:1,000 | Santa Cruz Biotech. |
| TLR2 | sc-21759 | 1:1,000 | Santa Cruz Biotech. |
| MyD88 | sc-74532 | 1:1,000 | Santa Cruz Biotech. |
| p-JNK | sc-6254 | 1:1,000 | Santa Cruz Biotech. |
| p-Akt | sc-393887 | 1:1,000 | Santa Cruz Biotech. |
| COX-2 | sc-376861 | 1:1,000 | Santa Cruz Biotech. |
| IL-1β | sc-4592 | 1:1,000 | Santa Cruz Biotech. |
| MMP9 | sc-393859 | 1:1,000 | Santa Cruz Biotech. |
| MMP2 | sc-13595 | 1:1,000 | Santa Cruz Biotech. |
| p-Smad-3 | sc-517575 | 1:1,000 | Santa Cruz Biotech. |
| TGF-β1 | sc-130348 | 1:1,000 | Santa Cruz Biotech. |
| KEAP1 | sc-365626 | 1:1,000 | Santa Cruz Biotech. |
| PTEN | sc-133197 | 1:1,000 | Santa Cruz Biotech. |
| Nrf2 | sc-365949 | 1:1,000 | Santa Cruz Biotech. |
| HO-1 | sc-136960 | 1:1,000 | Santa Cruz Biotech. |
| SOD1 | sc-271014 | 1:1,000 | Santa Cruz Biotech. |
| GPX4 | ab125066 | 1:1,000 | Abcam (Cambridge, UK) |
| αSMA | ab5694 | 1:1,000 | Abcam |
| Caspase-3 | CSB-PA05689A0Rb | 1:1,000 | Cusabio (Hubei, China) |
| TNF-α | 5178SC | 1:1,000 | Cell Signaling Tech. (Danvers, MA, USA) |
| p-Smad-2 | #3108 | 1:1,000 | Cell Signaling Tech. |
| Secondary anti-rabbit | 7074S | 1:5,000 | Cell Signaling Tech. |
| condary anti-mouse | 7076S | 1:5,000 | Cell Signaling Tech. |
